# Diverse Factors Affecting Efficiency of RNAi in Honey Bee Viruses

**DOI:** 10.3389/fgene.2018.00384

**Published:** 2018-09-11

**Authors:** Dahe Yang, Xiang Xu, Hongxia Zhao, Sa Yang, Xinling Wang, Di Zhao, Qingyun Diao, Chunsheng Hou

**Affiliations:** ^1^Institute of Apicultural Research, Chinese Academy of Agricultural Sciences, Beijing, China; ^2^Key Laboratory of Pollinating Insect Biology, Ministry of Agriculture and Rural Affairs of the People’s Republic of China, Beijing, China; ^3^Graduate School of Chinese Academy of Agricultural Sciences, Beijing, China; ^4^Guangdong Key Laboratory of Animal Conservation and Resource Utilization, Guangdong Public Laboratory of Wild Animal Conservation and Utilization, Guangdong Institute of Applied Biological Resources, Guangzhou, China

**Keywords:** RNAi, honey bee viruses, sensitivity of RNAi, gene function, *Apis mellifera*

## Abstract

Infection and transmission of honey bee viruses pose a serious threat to the pollination services of crops and wild plants, which plays a vital role in agricultural economy and ecology. RNA interference (RNAi) is an effective defense mechanism against commonly occurring viral infections of animals and plants. However, recent studies indicate that the effects of RNAi on the honey bee can induce additional impacts and might not always be effective in suppressing the virus. Moreover, the RNAi responses differed in relation to the developmental stage of the insect and the target tissue used, even though the same method of delivery was used. These results indicate that further analysis and field experiments should be performed to characterize the varying effectiveness of RNAi-based methods for treating honey bee viral infections. In this review, we provide an overview of the current knowledge and the recent progress in RNAi-based anti-viral treatments for honey bees, focusing in particular highlight the role of the dsRNA-delivery method used and its effect on RNAi efficiency and demonstrate the potential practical value of this tool for controlling the virus. We conclude studying the gene function and disease control of honey bee by RNAi technology requires a complex consideration from physiology, genetics to environment.

## Introduction

Honey bees are important pollinators of agricultural crops and ecological systems. The honey bee population in European and United States has rapidly deceased in the past few decades and the decrease was associated with microbial infections, parasitic infections, and other biotic or abiotic stress (Cox-Foster et al., 2007; Hou et al., 2014). Honey bee-infecting pathogens as a major impacts have caused severe economic losses by affecting pollination and bee colony population in agricultural and apicultural industry (Aizen et al., 2009). Among the honey bee pathogens, viruses are the majority factors impacted honey bee health but have been poorly characterized (Brutscher et al., 2016). Over 20 honey bee viruses have been identified, some of which cause chronic infection until the bees encounter other stress factors, such as infection with *Varroa destructor* (Shen et al., 2005; Di Prisco et al., 2011) or *Nosema* *ceranae* (Toplak et al., 2013).

Generally, covert infections of honey bee viruses were built in colony that shown no clinical symptoms under the no other stressors. However, there are still a few of viruses that can cause typical signs. Deformed wing virus (DWV), chronic bee paralysis virus (CBPV), black queen cell virus (BQCV), Israeli acute paralysis virus (IAPV), and sacbrood virus (SBV) can make honey bee display the visible symptoms such as deformed wing, paralyzed, black cell and pupae sacbrood. In addition, the viruses establish acute infections such as the infection caused by acute bee paralysis virus (ABPV), which produces apparent symptoms (Azzami et al., 2012). Thence, most of other viruses can be frequently detected in seemingly-health bees and cannot make an accurate conclusion through the phenotypic characteristics. Thus, molecular detection based on the polymerase chain reaction (PCR) technology becomes the conventional means for identifying the bee viruses.

However, although most of honey bee viruses can be detected by PCR, beekeepers can rarely take effectively measures to limit viral infections. Most of the honey bee viruses are positive-sense, single-stranded RNA viruses, which are primarily distributed into *Discitrovirus* family. The viruses from *Discitrovirus* family have been shown to readily establish persistent infections and cause large economic losses in the apicultural industry because these viruses are able to replicate efficiently by using internal ribosome entry sites (IRES)-mediated translation mechanism, which is different from the cap-dependent replication mechanism used by most other viruses (Fernández-Miragall et al., 2009). Thus, these viruses are not only difficultly found in host but also there are no effective strategies to control them. However, with the advent of RNA interference (RNAi)-based methodologies, there has been an increasing interest in assessing potential applications of RNAi in controlling virus-mediated diseases and agricultural pests in both laboratory and field (Miller et al., 2008; Hunter et al., 2010; Garbutt et al., 2013; Di Lelio et al., 2014).

In fact, most of insect immune responses are involved in antiviral mechanism of honey bee. Toll, Immune deficiency (Imd), c-Jun N-terminal kinase (JNK) and Janus kinase/Signal Transducer and Activator of Transcription (Jak-STAT) pathways have been confirmed that play a vital role in resistance against virus infection (Brutscher et al., 2015). In addition, several physiological defenses related with antiviral responses of honey bee including melanization, encapsulation, and antimicrobial peptides have been identified (Brutscher et al., 2015). Although all these immune responses contribute to antiviral action, RNAi is still the most broadly defense mechanism in honey bee (Niu et al., 2014).

**FIGURE 1:**
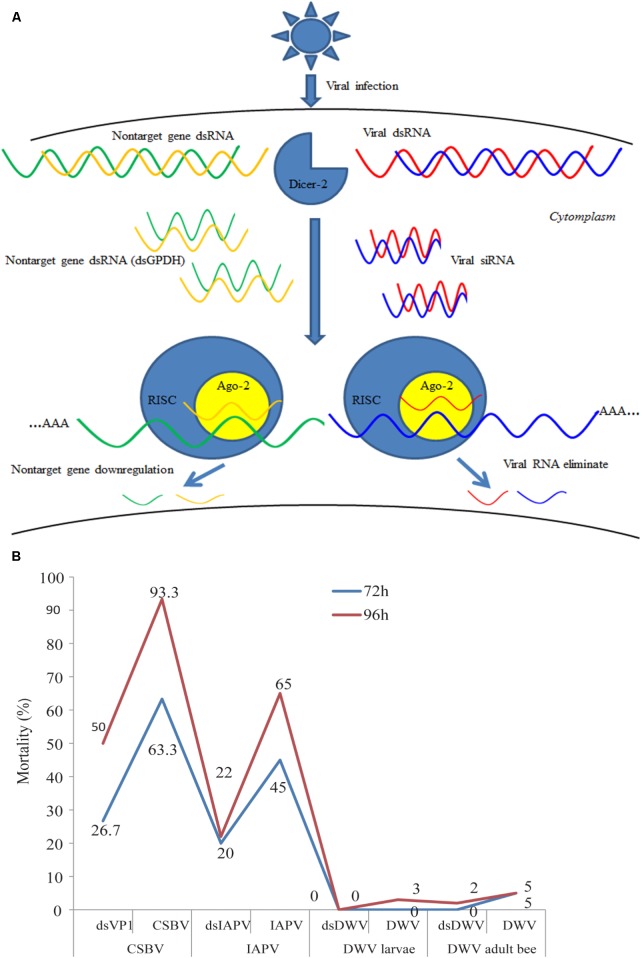
Overview of the process of RNAi-mediated gene silencing, possible off-target effects and the mortality of honey bee or larvae treated with RNAi against different honeybee viruses. **(A)** The short-interfering RNA (siRNA) pathway is one of the major ways for honey bee antiviral defense. Usually, the honey bee RNAi-pathway is induced by Dicer-like cleavage of viral dsRNA into siRNAs. In honey bees, non-specific dsRNA-mediated reduction in virus abundance (Flenniken and Andino, 2013) and degaradation on non-target genes (Jarosch and Moritz, 2012), but the mechanisms of this response have not been fully characterized. AGO2, Argonaute-2; RISC, RNA-induced silencing complex. **(B)** Bees or larvae were treated with viruses (CSBV, IAPV, and DWV) or target virus-double-stranded RNA (dsVP1, dsIAPV, and dsDWV). The number indicates the percentage of mortality treated with dsRNA and without for different viruses. CSBV, IAPV, DWV, and VP1 mean the Chinese sacbrood virus, Israeli acute paralysis virus, deformed wing virus, and virus protein 1 (Maori et al., 2009; Hunter et al., 2010; Liu et al., 2010; Desai et al., 2012).

RNAi was first discovered in transgenic plants (Mathieu and Watts, 1989), followed by the discovery of its prevalence in a wide range species (Mao et al., 2007; Miller et al., 2008; Tian et al., 2009; Garbutt et al., 2013; Ren et al., 2014). RNAi is the major mechanism of antiviral defense, which is a sequence specific and post-transcriptional gene silencing that is triggered by double stranded RNA (dsRNA) (**Figure 1A**) (Brutscher et al., 2015). RNAi can be applied to interfere with expression of intercellular genes, rendering it a potentially powerful tool for the development of novel insect virus control strategies (Liu et al., 2012). Direct evidence of antiviral function of RNAi has been reported in *Drosophila melanogaster* (van Rij et al., 2006). Genome analysis shown that honey bees encode RNAi machinery genes, such as dicer-like, Argonaute (Ago) 2 (Elsik et al., 2014). Experiment evidence confirmed that RNAi is systemic in honey bee and found that *sid-1* gene was essential for systemically administered dsRNA and gene silencing (Aronstein et al., 2006). RNAi has been used to study developmental gene expression of honey bee larvae (Jarosch et al., 2011; Kamakura, 2011; Wilson and Dearden, 2012), immunity of adults (Ament et al., 2012; Wang et al., 2012), and gene function of honey bee brain (Mustard et al., 2010; Hassani et al., 2012; Louis et al., 2012) as well as the functions of viral components such as the internal ribosome entry site within the intergenic region (IGR-IRES) (Au et al., 2017). In addition, dsRNA treatment has been also used to control honey bee parasites such as *N. ceranae* (Paldi et al., 2010), ectoparasitic mite *V. destructor* (Garbian et al., 2012; Campbell et al., 2016) and small hive beetle (Powell et al., 2016). More important, previous studies have demonstrated that RNAi can be used for controlling honey bee viruses and the success of using this treatment method indicates that RNAi could be potentially used for reducing economic losses caused by bee colony-infecting viruses around the world (Evans et al., 2009; Maori et al., 2009; Hunter et al., 2010; Liu et al., 2010; Desai et al., 2012). With the development of RNAi, the applications of dsRNA delivery into honey bees and other insects have been increasingly improved (Jarosch and Moritz, 2011; Jarosch et al., 2011; Hassani et al., 2012). Recently experimental evidence confirmed that RNAi immune response was triggered by *Dicer-2* when honey bees were infected by SBV (Fung et al., 2018).

## The Factors Affecting Efficiency of Rnai

Although the RNAi has been commonly used in honey bee viruses (**Table 1**), there are more challenges associated with dsRNA delivery in honey bees than in other insects due to the lack of bee cell cultivation system (Maori et al., 2009; Hunter et al., 2010; Liu et al., 2010; Desai et al., 2012; Chen et al., 2014; Zhang et al., 2016). The efficiency of RNAi delivery can be influenced by several factors, which can act alone or in combination. Some of the influencing factors include the life stage of the target insect, stability of the target gene, target tissue site, and dsRNA quantity (Flenniken and Andino, 2013). The oral delivery of dsRNA of a non-target gene, dsRNA-GFP, to honey bee larvae caused changes in expression level of approximately 1400 genes, which account for 10% honey bee genes (Nunes et al., 2013). Moreover, molecular mechanisms underlying the RNAi-based antiviral effect in honey bees have not been fully characterized, and little is known about the optimal RNAi delivery method for treating honey bees in different development stages, castle, and aims (Niu et al., 2014).

**Table 1:** RNAi-based control of honey bee viruses.

Virus	Delivery method	Range of target	Reference
IAPV^a^	Oral	IRES^d^	Maori et al., 2009
IAPV^a^	Oral	Unknown	Hunter et al., 2010
IAPV^a^	Oral	RdRp^e^	Maori et al., 2009
IAPV^a^	Oral	5′ terminal	Chen et al., 2014
DWV^b^	Oral	RdRp	Desai et al., 2012
CSBV^c^	Oral	Vp1^f^	Liu et al., 2010; Zhang et al., 2016

*^a^IAPV, Israel acute paralysis virus; ^b^DWV, deformed wing virus; and ^c^CSBV, Chinese sacbrood virus; IRES^d^, internal ribosome entry site; RdRp^e^, RNA-dependent RNA polymerase; Vp1^f^, virus protein 1.*

## The Way for Delivery of Rnai

The methods of dsRNA delivery can influence the success of RNAi treatment. The soaking way is suitable only for certain insect cells and tissues as well as for specific insects of developmental stages that readily absorb dsRNA from the solution, and therefore, it is rarely used (Scott et al., 2013). Typically, two primarily dsRNA delivery methods are used: orally or via injection. Both methods have been used to control honey bee disease, although new delivery methods are under development (Jarosch and Moritz, 2011; Jarosch et al., 2011; Hassani et al., 2012). RNAi uptake by cells can occur via passive or active pathways (Whangbo and Hunter, 2008). The responses of cell receptors to these two delivery methods are considerably different and lead to significant differences in effectiveness of the RNAi treatments. For example, injection of dsRNA into the body cavity of locust had a higher sensitivity than that induced by oral dsRNA administration, and four dsRNase in gut juice of the locust can affect the sensitivity of RNAi (Wynant et al., 2014).

For injection delivery, cuticular damage caused stimulates immune function which can further complicate the interpretation of the results (Katoch et al., 2013). In order to avoid or reduce the effects induced by sample manipulation or RNAi injection, Nunes and Simões (2009) used a non-invasive method by using a vitellogenin RNAi system that involved administration of dsRNA to second instar larvae of honey bee. The data indicated that about 60% of treated larvae could develop into adult stage and that approximately 90% of vitellogenin transcripts in worker bees were silenced as compared to those of the untreated control group. Even though the same method of dsRNA delivery was used, the RNAi responses differed.

Delivery methods of RNAi can yield false positive results. Although adult worker bees are highly sensitive to the used delivery method, invasive delivery methods (such as injection) can induce the anticipated responses, which could then activate cellular or humoral actions related to physiology and survival (Nunes and Simões, 2009; Flenniken and Andino, 2013). In addition, recent studies showed that the mortality rate of RNAi-treated honey bees was correlated to the type of dsRNA delivery methods used (rather than the presence of RNAi) and found that the bee mortality was caused by detrimental effects of tissue damage in embryos and larvae of honey bee (Amdam et al., 2003; Aronstein and Saldivar, 2005).

A study has shown that silencing of *vitellogenin* gene will cause the honey bee workers into extremely earlier forages and leading to behavior maturation (Antonio et al., 2008). To understand better the interaction between different genes, Wang et al. (2013) developed an injection protocol for knockdown the two genes simultaneously, vitellogenin (*vg*) and ultraspiracle (*usp*), and found that vg plays a key role among the vg, usp and juvenile hormone (JH) during the process of behavioral maturation. However, Dearden et al. (2009) tried to inject the dsRNA into embryos but not applied any genes in practice.

## The Difference of Target Tissues or Genes and Time Injected

RNAi application and efficacy remains variable between genes, organisms and life stages, even insect species. Moreover, gene knockdown efficacy varies in different insect species depending on transcript level of target gene, protein turnover rates and dsRNA uptake efficiency by cells or organs. For instance, the effects obtained by injection of dsRNA on *D. melanogaster* and *Manduca sexta* have only been achieved in hemocytes compared to other tissues (Scott et al., 2013). In mosquitoes, most tissues can be reached through injection of dsRNA but depending on genes and dose-dependent in central nervous system (Biessmann et al., 2010). The sensitivity and effectiveness of RNAi vary and depend on the intrinsic characteristics of the target species, as well as the site of target tissue (Xavier, 2010). A few of insects, including the desert locusts and red flour beetle, are amenable to systemic RNAi gene silencing (Miller et al., 2008; Wynant et al., 2014). In contrast, insects such as tobacco hornworm and silk moth are not amenable to systemic RNAi gene silencing (Miller et al., 2008; Xavier, 2010). In order to understand the factors influencing the varied responses amongst different tissues, a study on migratory locust (an agricultural insect pest) was conducted by injecting dsRNA and analyzing the responses in various locust tissues (Ren et al., 2014). The results showed that the locust ovaries were completely insensitive to dsRNA. While further study showed that the injected dsRNA was absent in the follicle cells and oocytes and, the lack of uptake may be the primary factor for the ineffective RNAi response in locust ovaries. These findings reveal the tissue-dependent variability in responses to RNAi.

Although RNAi-based methods are commonly used to conduct functional studies of genes, the responses to RNAi treatments drastically vary among different species and tissues. As described by Mutti et al. (2011), they applied RNAi to knockdown the insulin receptor substrate (IRS) and target of rapamycin (TOR) in larvae reared on queen diet to investigated how the nutrition and JH signaling determine the caste of honey bee, and the results showed that knockdown the IRS and TOR will induce the different additional effects in transcriptome, proteome, and total lipid level. Analysis of the systemic effect of RNAi on honey bee demonstrated that abdominal application (injection) of small interfering RNA (siRNA) resulted in gene silencing of primarily the fat body tissue and the other tissue was not amenable to the RNAi treatment with this delivery method (Wang X.B. et al., 2010; Jarosch and Moritz, 2011). Similarly, hemocytes of *D. melanogaster* have been shown to have lower sensitivity to dsRNA than that shown by fat body (Miller et al., 2008; Xavier, 2010). While, when employed RNAi to knock down the DNA methyl-transferase 3 of honey bee, it caused wide and diverse changes in fat tissue (Libyarlay et al., 2013).

Evaluation of the effect of RNAi treatment at the mRNA and protein expression levels showed that the level of gene suppression by RNAi was directly influenced by the quantity of dsRNA used and the circadian rhythm of the bees (Katoch et al., 2013). The dsRNA injected into the hemolymph relies on the circulation system to carry them to the target sites. However, hemolymph has a heavy impact on the dsRNA and the impact varies amongst different species and target tissues. For example, significant reduction in silencing of Relish in honey bee heads showed that the silencing effect of dsRNA in tissues was discontinuous at the site of injection, abdominal hemocoel (Schlüns and Crozier, 2007). Apart from that, although the effects of RNAi treatment may be the same at the mRNA and protein level, the dsRNA injected will have the effect only in the morning (not evening) at the protein level (Leboulle et al., 2013).

The difference in susceptibility to degradation of dsRNA may be influenced by the size and quantity of dsRNA. A previous study demonstrated that the RNAi efficiency of long dsRNA (>69 bp) was higher than that of short dsRNA (31 bp) (Miller et al., 2012). In addition, the effect of RNAi is dose dependent. Wang et al. (2013) found newly emerged honey bee could well-accept 4 μL dsRNA, while the mortality rapidly increased when more than 4 μL dsRNA was injected. They suggested two or more days injection strategy may be more suitable than the single injection for an experiment which requires higher amount of injection volume. Although there were no reports about the efficiency of the old adult bees feed with dsRNA, emerging bees are used usually to perform RNAi experiment after artificially infected by certain virus, which means that the immunity response is determined on a relatively short period (Smet et al., 2016). As shown in **Figure 1B**, there was a significant difference in the mortality rate of virus-infected honey bees and virus-infected larvae after treatment with dsRNA against different viruses. Particularly, the mortality of honey bees treated with CSBV was 63.3% after 72 h post-treatment, whereas the mortality rate of DWV was 0% (Maori et al., 2009; Hunter et al., 2010; Liu et al., 2010; Desai et al., 2012). Therefore, even though the effects of different sizes of dsRNA have not been identified in honey bee, further investigations have to be made. Thus, several studies have reported the tissue-dependent variability in effectiveness of RNAi-mediated gene silencing and the findings are summarized in **Table 2**.

**Table 2:** The effectiveness of RNAi treatment in honey bee tissues.

dsRNA	Target gene	Delivery method	Target tissue	Effective (yes or no)	Reference
dsSID-1	amSid-1	Soaking	Transmembrane protein	Yes	Aronstein et al., 2006
dsGPDH	amGPDH	Injection	Ovary	No	Jarosch and Moritz, 2012
dsGPDH	amGPDH	Injection	Fat body	Yes, but also affects amSID-1, amATF-2, amDHAP-AT, and amCPR	Jarosch and Moritz, 2012
dsGFP	amGPDH	Injection	Ovary	No	Jarosch and Moritz, 2012
dsVG	amGPDH	Injection	Fat body	amCPR	Jarosch and Moritz, 2012
dsGFP	amGPDH	Injection	Fat body	amGPDH	Jarosch and Moritz, 2012
dsVG	amVg	Injection	Fat body	Yes	Wang et al., 2013
dsUSP	amUsp	Injection	Fat body	Yes	Wang et al., 2013
dsDNMT3	amDNMT3	Oral	Whole body	Yes, but also affects ES, IR, ATE, AEB	Libyarlay et al., 2013

*dsGPDH, glycerol phosphatedehydrogenase; SID-1, systemic RNA interference defective; ATF, activating transcription factor; DHAP-AT, dihydroxyacetone- phosphate acyltransferase; dsVG, vitellogenin; CPR, cytochrome P450 reductase; dsGFP, double strand green fluorescent protein; dsDNMT3, double strand DNA methyl-transferase 3; ES, exon skipping; IR, intron retention; ATE, alternative terminal exon; AEB, alternative exon boundary; USP, ultraspiracle.*

## Potential Effects of Other Honey Bee Pathogens

Some pathogens of honey bee will possibly impact expected results. Experiment studies have confirmed that seemingly healthy bees can harbor several diseases, including viral infections (Todd et al., 2007). For example, Chen et al. (2004) revealed that a large number of emergent honey bees were simultaneously infected by multiple viruses such as DWV, SBV, and Kashmir bee virus (KBV). Moreover, when inoculating mix of several viruses of IAPV, SBV, KBV, DWV, and BQCV to cell and adult bees, the results showed that IAPV was rapidly increase to higher level than others even SBV was the main component of the mixture (Carrillo-Tripp et al., 2016). In addition, bees often harbor mixed infections caused by several viruses along with other pathogens such as *Nosema apis* (Todd et al., 2007). Thus, other pathogens might cause unexpected results. For example, RNAi was used to silence prophenoloxidase, which was considered as a resistance to American foulbrood (AFB), and found that no difference between RNAi treated and untreated groups (Chan, 2012). In addition, the viruses are not easily been detected and leading to unexpected results if they built covert infection at lower level (de Miranda et al., 2010). Therefore, the effectiveness of RNAi treatment against viral infections may be reduced by the prevalence of other pathogens or stresses.

## Suppression of Viral Rnai Suppressor

Some plant and animal viruses have developed an effective strategy during the course of evolution with the host. For example, *Cucumber mosaic virus* has been shown to encode a 2b suppressor that inhibits *Arabidopsis* Ago1 cleavage activity to counter plant defense (Zhang et al., 2006). Furthermore, suppressors, including 2b, not only bind Ago protein but can also bind dsRNA and siRNA *in vitro* (Wang et al., 2006; Wang Y. et al., 2010). Subsequently, 1A, an insect virus suppressor of *Cricket Paralysis virus* (CrPV), was shown to bind to Ago-2 to inhibit slicing of mRNA *in vitro* (Nayak et al., 2010). In addition, virus suppressors, such as P6 of *Cauliflower mosaic virus* and B2 of *Flock house virus*, also bind other proteins or RNA components of RNAi to inhibit the RNAi (Haas et al., 2008; Ruiz-Ferrer and Voinnet, 2009). Based on analysis of viral suppressor of RNAi (VSR) of *Drosophila* C virus and CrPV, DvExNPGP is representative majorly conserved motif of *Dicistroviridae* family, which has the ability to express virus suppressor protein (van Rij et al., 2006; Nayak et al., 2010). Likewise, sequence analysis showed that several honey bee viruses including IAPV, KBV and ABPV, also contain a DvExNPGP motif at the 5′ terminus of their genomes, and demonstrated these honey bee viruses might encode a VSR and experiment confirmed the level of IAPV was reduced when silenced IAPV-encoded putative suppressor of RNAi (Chen et al., 2014). Apart from virus suppressors, other mechanisms that enable interference with RNAi and prevent spread of RNA-mediated defense signal have also been identified. For example, p25, a viral movement protein of potato virus X, has been characterized as an effector suppressing anti-viral, the possibility should not be dismissed (Voinnet et al., 2010).

## Possible Affects From Genetically Modified Plants

The energy resource of honey bee is major from flowering plants, fruits, or crops and wild plants secreted honeydew. However, genetically modified plants and animals are being increasingly used for pest control or disease prevention. A number of novel approaches for RNAi-based pest control for plants have also been studied (Tian et al., 2009; Li et al., 2011; Zhu et al., 2011). To identify the potential effects of Bt crops, Vélez et al. (2016) employed the dsRNA of *Diabrotica virgifera virgifera* ATPase and found that RNAi had still impact on larval development and adult life span of honey bee, although there was no significant difference between treatment and control groups. However, despite the development of transgenic plants by using RNAi seems promising, the effect of the transgenic plants on honey bees has not been fully characterized. Moreover, the effect of genetically modified plant components on the dsRNA delivered to the honey bees is also poorly understood.

## Future Perspectives

Although the uses of RNAi for controlling viruses hold a significant promise, it is still in its infancy in honey bee and has its limitations and possible risk (Burand and Hunter, 2013). Multiple virus infection is very common in honey bee colonies even in one bee (de Miranda et al., 2010). Different virus strains or highly similar viruses in genome could be present at the same time in such field isolates as DWV and *Varroa destructor virus* (VDV), or among IAPV, Kashmir bee virus (KBV) and Kakugo virus (KV). Even if purified virus was from experimental infection honey bee samples, it still might host several viruses (Carrillo-Tripp et al., 2016). Therefore, vsiRNAs from siRNA pathway of various viruses can be produced. In addition, it is still unknown about siRNA response of multiple virus infection because there are no infectious clones for single virus to use (Niu et al., 2014). Therefore, it might not get exactly the expected results from siRNA pathway in bees and progress to impact the use of dsRNA in beekeeping practice.

Although considerable progress has been achieved in developing RNAi-based treatments for controlling honey bees viruses, several important questions remain to be answered. First, RNAi-based approaches should include utilization of next generation sequencing technology and the methodology used to identify novel potential target genes (Wang et al., 2011). Previous studies have demonstrated that dsRNA can produce off-target effects that have physiology, developmental, and reproductive consequences in the target organism (Jarosch and Moritz, 2012).

The analysis of honey bee hemolymph components in detail is essential to design an effective RNAi strategy. The stability of dsRNA in the target insects may vary due to the differences in the types of extracellular enzymes secreted into various organs. For example, DNAse/RNAse activity in lepidopteran species can affect the RNAi effectiveness (Liu et al., 2010; Allen and Walker, 2012). In addition, dsRNA was rapidly degraded after it was injected into *M. sexta*, whereas dsRNA injected in *B. germanica* persisted for a longer time period (Garbutt et al., 2013). This gap can be alleviated by systematic analysis of molecular physiological basis of RNAi mechanisms in honey bee will facilitate the application of RNAi for resolve of gene function.

Although a number of studies have been performed to assess the application of RNAi in honey bees, the efficiency of gene silencing through the various developmental stages of the honey bee have not been thoroughly characterized. The type of target tissue/organ and the specific development stage in which RNAi responses are obtained indicate not only the characteristics of the examined genes, but can also indicate the functional and developmental role of the target genes. Typically, RNAi is used to target the following three insect developmental stages: egg, larva, and adult. The developmental stage used for RNAi treatment may result in varying responses. For example, injection of dsRNA in pupae and adults of *Athalia rosae* lead to higher RNAi treatment efficiency than that obtained by using eggs, and the results showed that application of dsRNA via injection into the mid to late larval stages did not yield different results (Yoshiyama et al., 2013). Furthermore, another study showed that RNAi treatment begins to have effects in the larvae infected by CSBV of *Apis cerana* 12 h after oral application of dsRNA (Liu et al., 2010).

For systematic RNAi application, the size and quantity of dsRNA used should be considered. Based on the findings of the previous studies, we speculate that there may have been two factors that may have influenced the results of RNAi treatment in some studies. The first factor may have been inefficient dsRNA uptake or no response of intracellular RNAi machinery (Nunes and Simões, 2009). The second factor may be related to the optimum quantity of dsRNA that needs to be administered to the bees for obtaining gene silencing. Studies indicate that dsRNA uptake is inefficient in the ovaries of locust (**Table 2**). Since injection of dsRNA into honey bees is not a convenient and practical method, future research should focus on developing methods that enable efficient uptake of dsRNA by the target tissues and also enable the dsRNA to persist *in vivo* after oral application. In addition, study is required to address several questions, including the role and interactions of siRNA from other pathogens with the host RNAi machinery. Taken together, we conclude that much works have to be done to make the RNAi-based treatment strategy become reliably effective tool to study gene functions and gene mechanisms of honey bees.

## Author Contributions

CH and QD conceived this manuscript. DY, XX, HZ, SY, XW, and DZ participated in the writing, reviewing, and critical analysis of this manuscript. CH and QD coordinated the manuscript.

## Conflict of Interest Statement

The authors declare that the research was conducted in the absence of any commercial or financial relationships that could be construed as a potential conflict of interest. The handling Editor declared a shared affiliation, though no other collaboration, with several of the authors (DY, XX, SY, XW, DZ, QD, and CH).
